# The Supercooling Responses of the Solitary Bee *Osmia excavata* (Hymenoptera: Megachilidae) under the Biological Stress of Its Brood Parasite, *Sapyga coma* (Hymenoptera: Sapygidae)

**DOI:** 10.3390/insects13030235

**Published:** 2022-02-27

**Authors:** Zhuo Yan, Lina Wang, Gadi V. P. Reddy, Shimin Gu, Xingyuan Men, Yunli Xiao, Jianwei Su, Feng Ge, Fang Ouyang

**Affiliations:** 1 State Key Laboratory of Integrated Management of Pest Insects and Rodents, Institute of Zoology, Chinese Academy of Sciences, Beijing 100101, China; yanzhuo@ioz.ac.cn (Z.Y.); gushimin@ioz.ac.cn (S.G.); sujw@ioz.ac.cn (J.S.); 2 CAS Center for Excellence in Biotic Interactions, University of Chinese Academy of Sciences, Beijing 100049, China; 3 Applied Department, Weihai Wendeng Bigdata Bureau, Weihai 264200, China; lina_wang_o@163.com; 4 USDA ARS-Southern Insect Management Research Unit, 141 Experiment Station Rd., P.O. Box 346, Stoneville, MS 38776, USA; gadi.reddy@usda.gov; 5 Institute of Plant Protection, Shandong Academy of Agricultural Sciences, Jinan 250100, China; menxy2000@hotmail.com; 6 Plant Protection Department, Shandong Agriculture Technology Extension Center, 21 Minziqian Road, Jinan 250100, China; luckylily68@163.com

**Keywords:** overwintering, supercooling, solitary bee, brood parasite

## Abstract

**Simple Summary:**

Insects have different strategies to adapt to environment change and parasites, but the supercooling response of pollinator populations under the biological stress has not been sufficiently investigated. This study assessed the supercooling traits of the solitary bee and its brood parasite. We discovered significant differences in the supercooling points were found between the solitary bee and its brood parasite in the same sex, and also between sexes of same species. The supercooling traits (supercooling points, fresh weight, and fat content) of the two species were significantly positively correlated. Our results suggest that the supercooling points of the solitary bee increase under the biological stress of its brood parasite at a certain level. Overall, the supercooling of pollinator populations varies regularly under brood parasitism pressure.

**Abstract:**

(1) Background: Many insects have evolved different strategies to adapt to subzero temperatures and parasites, but the supercooling response of pollinator populations under the brood parasitism pressure has not been sufficiently investigated. (2) Methods: This study assessed the supercooling traits (supercooling points, fresh weight and fat content) of the solitary bee *Osmia excavata* Alfken and its brood parasite, *Sapyga coma* Yasumatsu & Sugihara. We measured 4035 samples (3025 *O. excavata* and 1010 *S. coma*, one individual as one sample) and discovered the supercooling traits relations between solitary bee and brood parasite. (3) Results: Significant differences in the supercooling points were found between *O. excavata* (females: −24.18 (−26.02~−20.07) vs. males: −23.21 (−25.15~−18.65) °C) and *S. coma* (females: −22.19 (−25.46~−18.38) vs. males: −20.65 (−23.85~−16.15) °C, *p* < 0.0001) in the same sex, and also between sexes of same species. The two species’ supercooling traits (supercooling points, fresh weight, and fat content) were significantly positively correlated. The supercooling points of the solitary bee varies regularly under brood parasitism pressure. (4) Conclusions: Our study indicates the supercooling traits relationships between a solitary bee and its brood parasite and suggests that the supercooling points of the solitary bee increase under the biological stress of its brood parasite in a certain level.

## 1. Introduction

Overwintering animals, especially insects, have evolved different strategies to adapt to low-temperature environments in winter [1]. The two main strategies used by insects to survive subzero temperatures included freeze-avoidance and freeze-tolerance [2]. The supercooling point is an overwintering indicator that describes the cold-resistant ability of insects [3]. When the body temperature reaches a specific low temperature, body fluids begin to crystallize. That low temperature is called the supercooling point [1]. Supercooling processes can prevent body fluids from freezing when the temperature is below its equilibrium freezing point. Such supercooling processes occur widely in many types of animals, including insects, amphibians, snails, and reptiles [4,5,6,7]. Insect supercooling occurs during overwintering at subzero temperatures [8]. The overwintering capacity of some insects can be influenced by environmental conditions, including different temperatures, temperature durations, and thermoperiodicity, which is called cold tolerance plasticity [9,10,11]. Supercooling points of insects are affected by multiple intrinsic and extrinsic factors, including the accumulation of glycerol, polyhydric alcohols, trehalose, sorbitol, mannitol, and other low molecular substances, or the reduction of water content and glycogen concentrations [12,13,14]. Additionally, supercooling points may be affected by the insect’s life stage (feeding or non-feeding stage), the ambient temperature, and the individual’s exact instar [7]. Despite recent progress, there is not enough research on supercooling points or overwintering capacity affected by brood parasites.

In a forecast of pollinator responses to climate change, floral host plants (i.e., bottom-up processes) and natural enemies (i.e., top-down forces) both play key roles in pollinator population regulation [15]. Warming temperature on account of climate change has direct and indirect fitness effects on solitary bees [16]. Although insects overwintering [17,18,19,20,21] and host-parasite relations [22,23,24,25,26] have been extensively investigated, the overwintering and supercooling response of pollinator populations under the brood parasitism pressure in cold winter has not been sufficiently investigated.

The goal of this study was to determine the relationship of supercooling traits between the solitary bee *Osmia excavata* Alfken (Hymenoptera: Megachilidae) [27] and its brood parasite *Sapyga coma* Yasumatsu & Sugihara (Hymenoptera: Sapygidae) [26]. The solitary bee *O. excavata* is an important pollinator of fruits and vegetables that is widely distributed in northern China. *O. excavata* has one generation per year, and their adults feed on nectar and pollen [28]. These bees usually build their nests in reeds or holes in the walls of buildings. Females create multiple earthen cells from mud from the inside out in sequence and lay a single egg in each cell. Pre-reproduction individuals develop alone in their cells, through their egg, larval, pupal, and pre-reproductive adult stages (in cocoons) from April to March. New adults then tear open their cocoons and emerge to mate, feed, and reproduce after making new cells. In fall and winter, farmers collect reeds with cocoons, store them in a refrigerator at 0–4 °C, and use the bees to pollinate apple and cherry orchards in the spring [27]. *S. coma* is an important brood parasite (kleptoparasite) of *O. excavata* that lays eggs in unfinished cells of *O. excavata* while the solitary bee is away [26]. Larvae of *S. coma* result in the solitary bee’s larvae death. The two species enter diapause as non-emerged adults inside cocoons for a 7-month-overwintering period (September– March) in northern China [26]. In the orchards, the two species overwinter inside thick reeds, which together with their cocoons provide some insulation from cold air and ice present outside reeds. Only a few studies have investigated the supercooling points of *Osmia* bees or their natural enemies [29,30,31].

Therefore, exploring the potential supercooling response of the solitary bee *O. excavata* and its brood parasite *S. coma* provides an opportunity to investigate the differences and correlations of two different species that experience the same cold environment. In this study, we measured the supercooling points and some physiological parameters for several individuals within the species and attempted to find some correlations. Our specific goals of this study are three-fold: (1) we identify associations between the supercooling points of the solitary bee and its brood parasite; (2) we transparentize relationships of physiological parameters between the solitary bee and its brood parasite; (3) we establish whether supercooling responses of the solitary bee vary under the biological stress of its brood parasite.

## 2. Materials and Methods

### 2.1. Experimental Insects

The solitary bee *O. excavata* and its brood parasite *S. coma* were obtained by collecting reeds, in which they build nests, from fifty villages in the north part of the Jiaodong Peninsula district (35°05′~37°50′ N, 119°16′~122°42′ E), Shandong Province, China. All the *O. excavata* were the offspring of released individuals under semi-manual management in 2017 spring. Most *S. coma* were wild populations. Twenty reed tubes full of nested cells were collected from each village in December 2017 and moved to Jinan City, then held indoors from December 2017 to January 2018 for the measurement. About thirty male and thirty female *O. excavata* adults were selected randomly from those reed tubes collected in each village. All individuals of *S. coma* from samples were collected for use in experiments because of their lower abundance (0~19.72% of 50 villages). All 4035 insects collected as described above (1507 females and 1518 males of the solitary bee, 527 females and 483 males of the parasite) were tested for supercooling points and physiological parameters, but some invalid data because of damaged samples were removed from the dataset before analysis.

The reed tubes we collected were stored in cardboard boxes near an open window to keep the storage temperature similar to that outside. The mean outside temperature was 2.5 °C in December 2017 and was −0.7 °C in January 2018 (data from Jinan City). After emergence from the ripped cocoons artificially (with scissors), insects were promptly weighed to obtain their fresh weight before the activities and defecation inside the laboratory of which the constant temperature is 23~26 °C, and each individual was then transferred into a refrigerator in 4 °C for 24 h before the determination of the supercooling point. We made sure all the samples were measured and stored in a same way throughout the whole experiment.

### 2.2. Measurement of Supercooling Points in Our Test Insects

The supercooling points we measured were determined at the Institute of Plant Protection, Shandong Academy of Agricultural Sciences. The supercooling determinator (SUN-II intelligent insect-SCP determinator, SUN Company, Jinan, China) was used to determinate the supercooling point (SCP) and the freezing point (FZP) of each individual. The sampled insects were fixed to the probes of the supercooling determinator using parafilm. Since the supercooling determinator has twenty probes, a group of nineteen samples and one blank control were assessed at one time. Each group, with the probes attached, was placed in a −80 °C freezer. Insects body temperature began to be recorded before being placed in the freezer and recorded 1.6 s apart by the determinator. Each group was recorded at the same time. Temperature recording continued until there were no further rises in temperature.

### 2.3. Measurement of Body Weight, Water Content, and Fat Content

The fresh body weight (abbreviated as FW) and the dry body weight (abbreviated as DW) were measured with an analytical balance (BSA224S analytical balance, Sartorius Group, Beijing, China). Fresh body weight was determined upon emergence artificially (dissecting the cocoons with scissors to get the unbroken individuals) before defecation. After use in measurement of supercooling points (SCPs) and other variables, each individual was put into a drying oven at 60 °C for 72 h and then weighed to determine the dry weight (abbreviated as DW).

After measuring each individual’s dry weight (DW), we measured the lean dry weight (abbreviated as LDW) as follows. We added 2 mL of a 2:1 mixture of chloroform and methanol, then grounded the insect into a homogenate. The homogenate was centrifuged for 10 min (2600 g), and then the supernatant was removed. This procedure was repeated a second time using the residue. The final residue after this second cycle was put in a drying oven for 72 h at 60 °C, and weighed to determine the constant weight, namely the lean dry weight (LDW) [8,32].

Fat content (FC, see Equation (1)) were calculated as below:

FC = DW − LDW,(1)

### 2.4. Statistical Analyses

The data of the solitary bee *O. excavata* and its brood parasite *S. coma* were organized using Microsoft Excel 2019. The comparisons among three or more sets of data were analyzed using the Mann–Whitney test and Kruskal–Wallis test, followed by Dunn’s multiple comparisons test with *p* < 0.05, while two sets of data were analyzed using Kruskal–Wallis test (two groups) by non-parametric test in Graphpad Prism 8.4.0. The correlations of supercooling points and physiological parameters (body weight and fat content) between *O. excavata* and *S. coma* were analyzed using Pearson correlation coefficient with *p* < 0.05, followed by simple linear regression in Graphpad Prism 8.4.0. We used generalized additive models (GAM) to examine the relations between supercooling points of *O. excavata* and *S. coma* parasite ratio, because this allowed us to relax the assumption that changes of supercooling points of *O. excavata* when the *S. coma* parasite ratio increased were linear [33]. We modeled the relations by smooth functions through a Gaussian distribution with an identity link (gam () in mgcv, version 1.8-38, Wood, 2021). All the figures were drawn using Adobe Illustrator 2020.

## 3. Results

### 3.1. The Supercooling Points of O. excavata and S. coma

**Figure 1:**
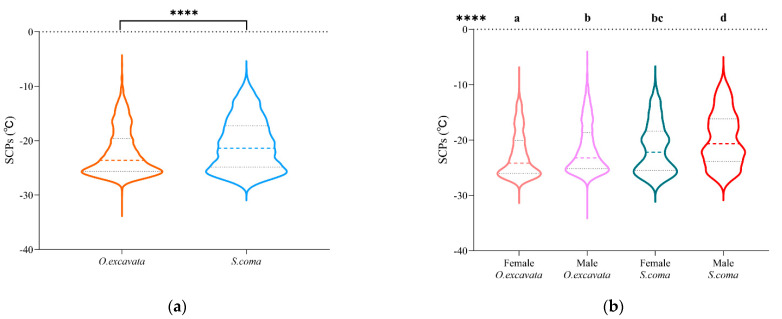
The distribution and difference of supercooling points (SCPs) between the solitary bee *O. excavata* and the brood parasite *S. coma* from 50 villages collected in December 2017, in the north part of the Jiaodong peninsula district, Shandong Province, China. The four asterisks denote significant difference (*p* < 0.0001) using the Mann-Whitney test (**a**) and significant difference (*p* < 0.0001) using the Kruskal-Wallis test (**b**). Bars with different superscripts denote significant differences (*p* < 0.0001) using Dunn’s multiple comparisons test (**b**). The dashed lines indicate medians in the middle and quartiles on both sides of the violin plots.

Taken together, the results from Figure 1 showed the solitary bee *O. excavata* had a lower supercooling point than the brood parasite *S. coma,* and females had a lower supercooling point than males within a species. The supercooling point was significantly lower in *O. excavata* than in *S. coma* (Median (first quartile~third quartile): −23.62 (−25.62~−19.60) vs. −21.37 (−24.83~−17.26) °C, *p* < 0.0001) (Figure 1a). The supercooling point differed significantly between the different sexes (within a species) and between the two species (within the same sex) (H = 176.4, *p* < 0.0001) (Figure 1b).

The supercooling point was significantly lower in the female than in the male *O. excavata* (−24.18 (−26.02~−20.07) vs. −23.21 (−25.15~−18.65) °C, *p* < 0.0001). The supercooling point was significantly lower in the female than in the male *S. coma* (−22.19 (−25.46~−18.38) vs. −20.65 (−23.85~−16.15) °C, *p* < 0.0001). Comparing across species, the supercooling point was significantly lower in the female *O. excavata* than *S. coma* (−24.18 (−26.02~−20.07) vs. –22.19 (−25.46~−18.38) °C, *p* < 0.0001), and, for males, the supercooling point was significantly lower in *O. excavata* than *S. coma* (−23.21 (−25.15~−18.65) vs. −20.65 (−23.85~−16.15) °C, *p* < 0.0001) (Figure 1b).

### 3.2. Relationship of Supercooling Points between O. excavata and S. coma

**Figure 2:**
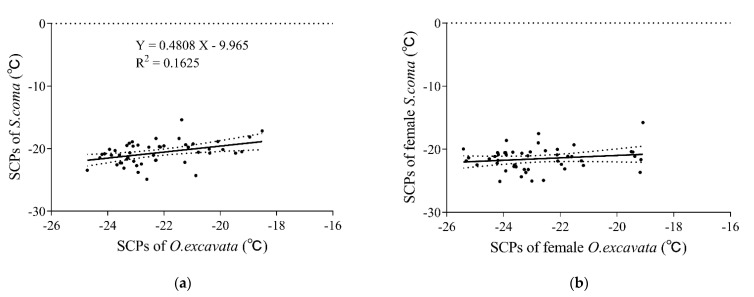

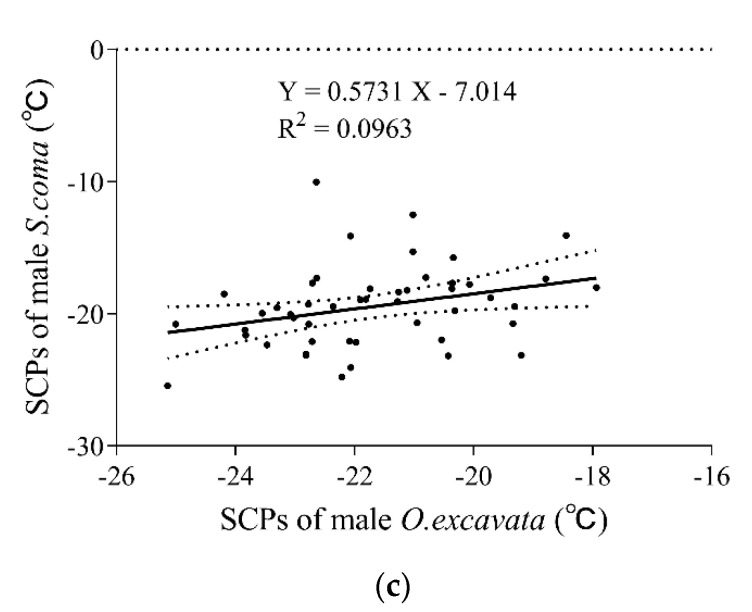
Influence of SCPs of *O. excavata* on SCPs of *S. coma* in (**a**) general, (**b**) females, and (**c**) males. Points show the raw data. Solid lines represent the result of the general linear model, while dashed lines represent the 95% confidence bands. Figures with a regression equation denote a significant (*p* < 0.05) linear regression and without otherwise.

Overall, the supercooling points of *S. coma* and *O. excavata* were significantly positively correlated (*r* = 0.4031, *p* = 0.0041, *n* = 49) (Figure 2a). The supercooling points of *S. coma* significantly increased with that of *O. excavata* increasing (*F*_(1, 47)_ = 9.120, *R*^2^ = 0.1625, *p* = 0.0041) (Figure 2a). The supercooling points of female *S. coma* and female *O. excavata* were not significantly positively correlated (*r* = 0.1761, *p* = 0.2260, *n* = 49) (Figure 2b). The supercooling points of male *S. coma* and male *O. excavata* were significantly positively correlated (*r* = 0.3103, *p* = 0.0300, *n* = 49) (Figure 2c). The supercooling points of male *S. coma* significantly increased with that of male *O. excavata* increasing (*F*_(1, 47)_ = 5.008, *R*^2^ = 0.09629, *p* = 0.0300) (Figure 2c).

### 3.3. Physiological Parameters of the Solitary Bee and Its Brood Parasite

**Figure 3:**
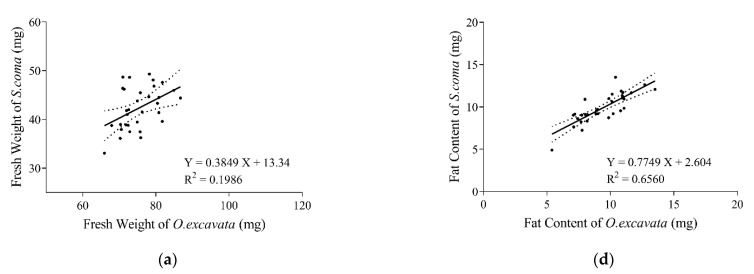

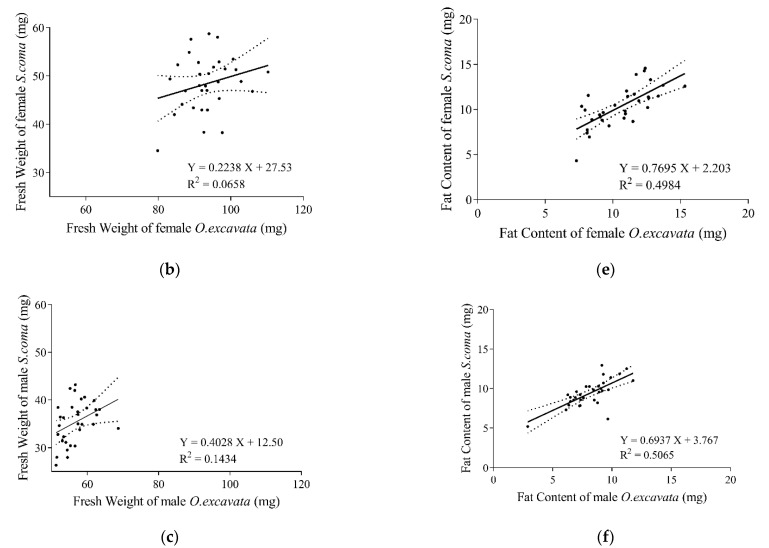
Influence of fresh weight of *O. excavata* on fresh weight of *S. coma* in (**a**) general, (**b**) females, and (**c**) males. Correspondingly, the influence of fat content in (**d**) general, (**e**) females, and (**f**) males, Points show the raw data. Solid lines represent the result of the general linear model, while dashed lines represent the 95%, confidence bands. Figures with a regression equation denote a significant (*p* < 0.05) linear regression and without otherwise.

In total, the fresh weights of *S. coma* and *O. excavata* were significantly positively correlated (*r* = 0.4456, *p* = 0.0106, *n* = 32) (Figure 3a). The fresh weights of *S. coma* significantly increased with increasing fresh weights of *O. excavata* (*F*_(1, 30)_ = 9.120, *R*^2^ = 0.1986, *p* = 0.0106) (Figure 3a). The fresh weights of female *S. coma* and female *O. excavata* were not significantly positively correlated (*r* = 0.2565, *p* = 0.1564, *n* = 32) (Figure 3b). The fresh weights of male *S. coma* and male *O. excavata* were significantly positively correlated (*r* = 0.3787, *p* = 0.0326, *n* = 32) (Figure 3c). The fresh weights of male *S. coma* significantly increased with increasing fresh weights of male *O. excavata* (*F*_(1, 30)_ = 5.021, *R*^2^ = 0.1434, *p* = 0.0326) (Figure 3c).

Generally, the fat contents of *S. coma* and *O. excavata* were significantly positively correlated (*r* = 0.8100, *p* < 0.0001, *n* = 32) (Figure 3d). The fat contents of *S. coma* significantly increased with that of *O. excavata* increasing (*F*_(1, 30)_ = 57.22, *R*^2^ = 0.6560, *p* = < 0.0001) (Figure 3d). The fat contents of female *S. coma* and female *O. excavata* were significantly positively correlated (*r* = 0.7060, *p* < 0.0001, *n* = 32) (Figure 3e). The fat contents of male *S. coma* significantly increased with that of male *O. excavata* increasing (*F*_(1, 30)_ = 29.81, *R*^2^ = 0.4984, *p* < 0.0001) (Figure 3e). The fat contents of male *S. coma* and male *O. excavata* were significantly positively correlated (*r* = 0.7117, *p* < 0.0001, *n* = 32) (Figure 3f). The fat contents of male *S. coma* significantly increased with that of male *O. excavata* increasing (*F*_(1, 30)_ = 30.79, *R*^2^ = 0.5065, *p* < 0.0001) (Figure 3f).

### 3.4. Relationship between Parasite Ratio and Supercooling Points of O. excavata

**Figure 4:**
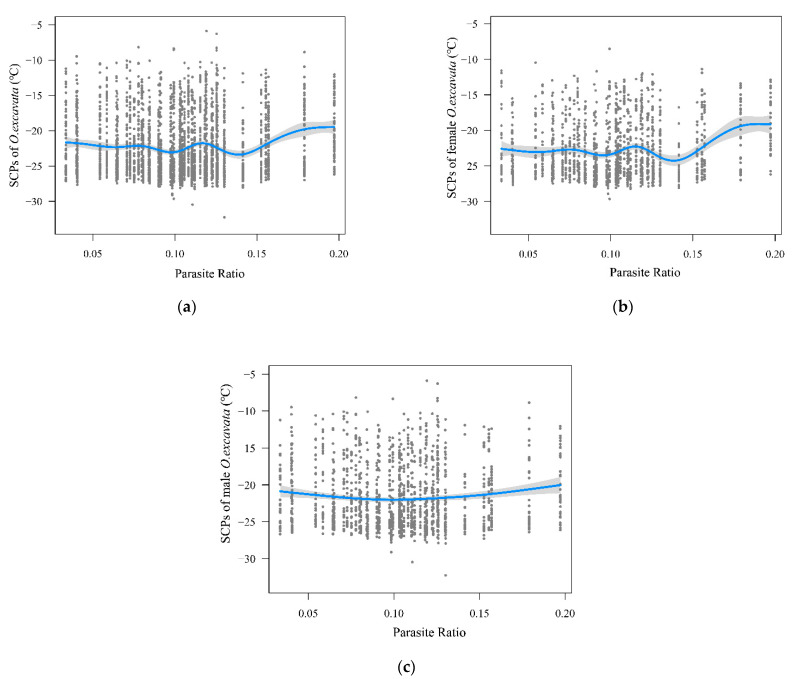
Influence of parasite ratio on SCPs of *O. excavata* in (**a**) general, (**b**) females, and (**c**) males. Points show the raw data. Blue lines represent the result of the generalized additive model. The shaded areas represent the 95% confidence interval.

Generalized additive model results showed a significant affected by parasite ratio to supercooling points of *O. excavata* (Figure 4). In total, the supercooling points of *O. excavata* and parasite ratio were significantly positively correlated (*r* = 0.0551, *p* = 0.0027, *n* = 2966). The supercooling points of *O. excavata* were non-linearly related to the parasite ratio. The GAM model explained 2.58% of the *R*^2^, and 2.86% of the deviance (*p* < 0.001). The supercooling points of *O. excavata* was in a fluctuant downward trend slightly before a parasite ratio of approximately 0.14, then obviously increased with a parasite ratio of approximately 0.14 (Figure 4a).

The supercooling points of female *O. excavata* and parasite ratios were significantly positively correlated (*r* = 0.0924, *p* = 0.0004, *n* = 1479). The supercooling points of female *O. excavata* were non-linearly related to parasite ratio. The GAM model explained 4.32% of the *R*^2^, and 4.85% of the deviance (*p* < 0.001). The supercooling points of female *O. excavata* was in a fluctuant downward trend slightly before a parasite ratio of approximately 0.14, then obviously increased with a parasite ratio of approximately 0.14 (Figure 4b).

The supercooling points of male *O. excavata* and parasite ratios were not significantly positively correlated (*r* = 0.0220, *p* = 0.3962, *n* = 1487). The supercooling points of male *O. excavata* were non-linearly related to parasite ratio. The GAM model explained 1.06% of the R^2^, and 1.24% of the deviance (*p* = 0.001). The supercooling points of male *O. excavata* were slightly downward before a parasite ratio of approximately 0.10, then obviously increased with a parasite ratio of approximately 0.10 (Figure 4c).

## 4. Discussion

In the current study, the interactions of several overwinter parameters between the solitary bee *O. excavata* and its brood parasite *S. coma* were found, following the analysis of a large numbers of individuals. Previous research recorded that two other species of *Osmia* were freeze-avoidance species [34]. We found that, due to its supercooling range, the overwintering strategy of the solitary bee *O. excavata* might be freeze avoidance. Liu et al. gives the supercooling points of male and female *O. excavata* as −24.46 °C and −25.02 °C, which is close to our results [35]. Comparing the two species, the supercooling points of *O. excavata* were significantly lower than those of *S. coma*. *O. excavata* might withstand lower ambient temperatures than *S. coma* with lower supercooling points. This might help *O. excavata* overcome colder winter. We also observed that the supercooling points of females of *O. excavata* and *S. coma* were lower than those of their males, suggesting that males may survive winters less well than females. In trap-nesting Hymenoptera, the parental generation controls the sex ratio of its offspring to adjust the female density to the available level of natural resources [36]. Given that the males’ investment in reproduction is only to mate, while females must also locate suitable nest sites, build nesting structures, and reproduce, lower parental investment in males (smaller body mass) compared to females may be justified [36]. Small body mass typically reduces an individual’s overwintering capability [37].

The supercooling points of *S. coma* increased with *O. excavata* increasing (Figure 2). We also found that the fresh weight and fat content of *S. coma* increased with *O. excavata* increasing. This may be because *S. coma* gained all nutrition from *O. excavata* in the larva period [26]. Solitary bees rely on a fixed energy budget during the overwintering period, during which they consume the nectar and pollen provision as larva from the parental generation [38,39]. Fresh weight [40] and fat content [41] were important physiological parameters of insects in overwintering periods.

Moreover, larger and heavier bees emerged earlier in spring than small and light conspecifics that experienced the same overwintering temperatures, which may increase bee fitness [30]. Larger body mass means greater nutrient reserves, improving survival during cold periods [37]. Our results show reasonably good agreement with these studies. As a kleptoparasite (also as a brood parasite) of *O. excavata*, *S. coma* kept a related to but different overwintering characteristic from its host through its evolution over time. Nevertheless, the relationship between supercooling point and these physiological parameters is very complex [42].

The incidence of parasitism was one of the critical restriction factors of reproductive output in solitary bee populations, indicating brood parasites play a key role in population regulation [26,43]. In our study, the supercooling points of *O. excavata* regular varied with an increasing *S. coma* parasite ratio in each community (including the two research species) from fifty sites (Figure 4). When the *S. coma* parasite ratio reached a certain level, the supercooling points of *O. excavata* stopped the slight fluctuate and obviously increased. In defense of natural enemies, solitary bees have evolved many adaptive traits. An empty cell in front of all brood cells was an effective measure to defend brood parasites [24,44]. Females of solitary bees can adjust the parental investment, which affects offspring size and the overwintering capacity when encountering resource constraints [44]. Because of the plenty of time consumed during *O. excavata* materials gathering activities [45], parasites might have the opportunity to lay eggs in solitary bee nests [26]. When the *S. coma* population increases to a certain number, to avoid losing the investment to parasites attacking the open cell, solitary bee females can limit each cell’s provision time [43]. With provision time reducing, nutrition in provision from a parental generation for each cell might decrease, resulting in an increase in the offspring’s supercooling points. Due to long-time absence from the nests [45], female *O. excavata* may have some opportunities to find the female *S. coma* in a low density, which may causes a slight fluctuate of supercooling points. When the high density of female *S. coma* causes a high parasite ratio (approximately >0.14), female *O. excavata* may have more opportunities to show an activity response, which may cause an increase of the supercooling points. As an overwintering indicator, higher supercooling points always mean worse cold-resistant ability, causing a population instability to the cold temperature [3]. Climate change and temperature increasing in winter may alleviate the population instability of solitary bees under the brood parasitism pressure, but this is a subject for further study.

Our study mainly shows that (1) the supercooling points of the brood parasite vary with the supercooling points of the solitary bee; (2) the physiological parameters relating to supercooling points vary with those of the solitary bee; (3) the supercooling points of the solitary bee increase under the biological stress of its brood parasite at a certain level.

## 5. Conclusions

Supercooling responses under biological stress of parasitism might exist in other insect species. The underlying reason for the appearance of the response in any particular species is ultimately unclear but is a suitable subject for further study. In the longer run, we believe that the ideas present in this paper may have broader application to explore the host supercooling responses of brood parasitism more deeply.

## Data Availability

The datasets generated during and/or analyzed during the current study are not publicly available due to degree related but are available from the corresponding author on reasonable request.
